# 1-{2-[(4-Hydr­oxy-3-methoxy­benzyl­idene)amino]eth­yl}-3-methylimid­azolium hexa­fluoro­phosphate

**DOI:** 10.1107/S160053680901928X

**Published:** 2009-05-29

**Authors:** Bin Li, Yi-Qun Li, Jie Liu, Wen-Jie Zheng

**Affiliations:** a Department of Chemistry, Jinan University, Guangzhou 510632, People’s Republic of China

## Abstract

In the title Schiff base salt, C_14_H_18_N_3_O_2_^+^·PF_6_^−^, the dihedral angle between the planes of the aromatic and imidazole rings is 24.84 (8)°. The mol­ecular structure exhibits an intra­molecular O—H⋯O hydrogen bond, which generates an *S*(5) ring motif. In the crystal structure, the cations and anions are connected *via* O—H⋯N and O—H⋯F hydrogen bonds, resulting in a trifurcated interaction for the phenolic H atom.

## Related literature

For bond-length data, see: Allen *et al.* (1987 ▶). For the synthesis of Schiff bases, see: Pradeep (2005 ▶); Butcher *et al.* (2005 ▶). For information on ionic liquids and their applications, see: Xiao *et al.* (2004 ▶); Welton (1999 ▶); Wilkes (2002 ▶).
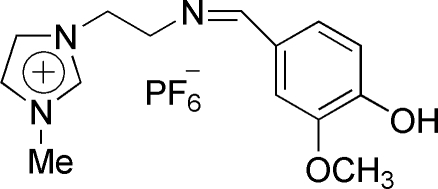


## Experimental

### 

#### Crystal data

C_14_H_18_N_3_O_2_^+^·PF_6_^−^
*M* *_r_* = 405.28
Monoclinic, 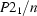

*a* = 7.5285 (10) Å
*b* = 12.6850 (16) Å
*c* = 17.827 (2) Å
β = 96.245 (2)°
*V* = 1692.3 (4) Å^3^
*Z* = 4
Mo *K*α radiation
μ = 0.24 mm^−1^
*T* = 173 K
0.48 × 0.43 × 0.38 mm

#### Data collection

Bruker SMART CCD area-detector diffractometer
Absorption correction: multi-scan (*SADABS*; Sheldrick, 1996 ▶) *T*_min_ = 0.894, *T*_max_ = 0.914
8519 measured reflections
3635 independent reflections
2904 reflections with *I* > 2σ(*I*)
*R*_int_ = 0.019

#### Refinement

*R*[*F* ^2^ > 2σ(*F* ^2^)] = 0.034
*wR*(*F* ^2^) = 0.099
*S* = 1.07
3635 reflections
238 parameters
H-atom parameters constrained
Δρ_max_ = 0.26 e Å^−3^
Δρ_min_ = −0.29 e Å^−3^

### 

Data collection: *SMART* (Bruker, 2002 ▶); cell refinement: *SAINT* (Bruker, 2002 ▶); data reduction: *SAINT*; program(s) used to solve structure: *SHELXS97* (Sheldrick, 2008 ▶); program(s) used to refine structure: *SHELXL97* (Sheldrick, 2008 ▶); molecular graphics: *SHELXTL* (Sheldrick, 2008 ▶); software used to prepare material for publication: *SHELXTL*.

## Supplementary Material

Crystal structure: contains datablocks I, global. DOI: 10.1107/S160053680901928X/su2112sup1.cif

Structure factors: contains datablocks I. DOI: 10.1107/S160053680901928X/su2112Isup2.hkl

Additional supplementary materials:  crystallographic information; 3D view; checkCIF report

## Figures and Tables

**Table 1:** Hydrogen-bond geometry (Å, °)

*D*—H⋯*A*	*D*—H	H⋯*A*	*D*⋯*A*	*D*—H⋯*A*
O2—H2*A*⋯O1	0.84	2.20	2.6589 (16)	114
O2—H2*A*⋯N1^i^	0.84	2.48	3.1584 (18)	139
O2—H2*A*⋯F2^ii^	0.84	2.49	3.0487 (18)	125

Symmetry codes: (i) 
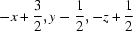
; (ii) 
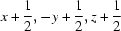
.

**Fig. 1.**
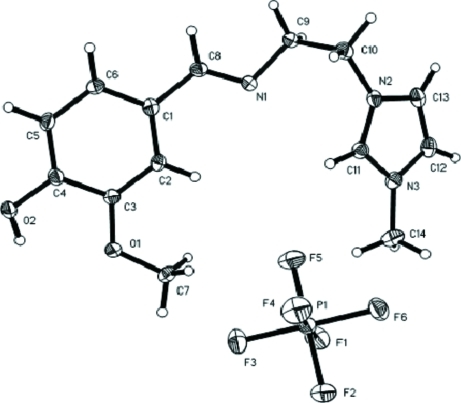
The molecular structure of the title compound, showing the atom numbering Scheme. Displacement ellipsoids are drawn at the 50% probability level.

## supplementary crystallographic information

### Comment

Ionic liquids have aroused considerable interest over the past decade due to their wide variety of properties such as high thermal and chemical stability, no measurable vapor pressure, non-flammability, friction reduction, and high loading capacity, *etc* (Xiao *et al.*, 2004; Welton, 1999; Wilkes, 2002). Schiff base compounds are one of the most prevalent mixed-donor ligands in the field of coordination chemistry (Pradeep, 2005; Butcher *et al.*, 2005). Our interest in this field of research lead us to synthesis the title compound, and we report here on the crystal structure of this novel ionic liquid-supported Schiff base.

The title compound is a Schiff base derived from the condensation of 4-hydroxy-3-methoxybenzaldehyde with the ionic liquid 1-(2-aminoethyl)-3- methylimidazolium hexafluorophosphate, under solvent-free conditions. The molecular structure of the title compound is illustrated in Fig. 1. The asymmetric unit comprises one cation and one PF_6_ anion. The bond lengths (Allen *et al.*, 1987) and angles are generally within normal ranges. The aromatic ring and imidazole ring are not coplanar but are inclined to one another by an angle of 24.84 (8)°. In the molecular structure, the intramolocular O2—H2A···O1 hydrogen bonds form a pseudo five membered ring [S(5) motif], thus locking the molecular conformation and eliminating any flexibility (Table 1).

In the crystal structure symmetry related cations and anions are connected via O-H···N and O-H···F hydrogen bonds (Table 1).

### Experimental

A mixture of the ionic liquid 1-(2-aminoethyl)-3-methylimidazolium hexafluorophosphate (4 mmol) and 4-hydroxy-3- methoxybenzaldehyde (3 mmol) was stirred for 4 h at rt, under solvent-free conditions. After completion of the reaction, ethanol (30 ml) was added to the reaction mixture. The solid product was then filtered off and washed with cold ethanol. The crude product was purified by recrystallization in ethanol/ethyl acetate(3:1 *v*/*v*). Single crystals, suitable for X-ray diffraction analysis, were obtained by slow evaporation of an ethyl acetate solution of the complex at rt.

### Refinement

All the H-atoms could be located in difference Fourier maps and were refined as riding atoms: O—H = 0.84 Å, with *U*_iso_(H) = 1.5 *U*_eq_(O); C—H = 0.95–0.98 Å with *U*_iso_(H) = 1.2 *U*_eq_(C).

### Crystal data

**Table:**

C_14_H_18_N_3_O_2_^+^·PF_6_^−^	*F*(000) = 832
*M**_r_* = 405.28	*D*_x_ = 1.591 Mg m^−^^3^
Monoclinic, *P*2_1_/*n*	Mo *K*α radiation, λ = 0.71073 Å
Hall symbol: -P 2yn	Cell parameters from 4600 reflections
*a* = 7.5285 (10) Å	θ = 2.3–27.1°
*b* = 12.6850 (16) Å	µ = 0.24 mm^−^^1^
*c* = 17.827 (2) Å	*T* = 173 K
β = 96.245 (2)°	Block, colorless
*V* = 1692.3 (4) Å^3^	0.48 × 0.43 × 0.38 mm
*Z* = 4	

### Data collection

**Table:**

Bruker SMART CCD area-detector diffractometer	3635 independent reflections
Radiation source: fine-focus sealed tube	2904 reflections with *I* > 2σ(*I*)
graphite	*R*_int_ = 0.019
φ and ω scans	θ_max_ = 27.1°, θ_min_ = 2.0°
Absorption correction: multi-scan (*SADABS*; Sheldrick, 1996)	*h* = −7→9
*T*_min_ = 0.894, *T*_max_ = 0.914	*k* = −16→13
8519 measured reflections	*l* = −22→22

### Refinement

**Table:**

Refinement on *F*^2^	Primary atom site location: structure-invariant direct methods
Least-squares matrix: full	Secondary atom site location: difference Fourier map
*R*[*F*^2^ > 2σ(*F*^2^)] = 0.034	Hydrogen site location: inferred from neighbouring sites
*wR*(*F*^2^) = 0.099	H-atom parameters constrained
*S* = 1.07	*w* = 1/[σ^2^(*F*_o_^2^) + (0.0489*P*)^2^ + 0.5604*P*] where *P* = (*F*_o_^2^ + 2*F*_c_^2^)/3
3635 reflections	(Δ/σ)_max_ = 0.001
238 parameters	Δρ_max_ = 0.26 e Å^−^^3^
0 restraints	Δρ_min_ = −0.29 e Å^−^^3^

### Special details

**Table:**

Geometry. All e.s.d.'s (except the e.s.d. in the dihedral angle between two l.s. planes) are estimated using the full covariance matrix. The cell e.s.d.'s are taken into account individually in the estimation of e.s.d.'s in distances, angles and torsion angles; correlations between e.s.d.'s in cell parameters are only used when they are defined by crystal symmetry. An approximate (isotropic) treatment of cell e.s.d.'s is used for estimating e.s.d.'s involving l.s. planes.
Refinement. Refinement of *F*^2^ against ALL reflections. The weighted *R*-factor *wR* and goodness of fit *S* are based on *F*^2^, conventional *R*-factors *R* are based on *F*, with *F* set to zero for negative *F*^2^. The threshold expression of *F*^2^ > σ(*F*^2^) is used only for calculating *R*-factors(gt) *etc*. and is not relevant to the choice of reflections for refinement. *R*-factors based on *F*^2^ are statistically about twice as large as those based on *F*, and *R*- factors based on ALL data will be even larger.

### Fractional atomic coordinates and isotropic or equivalent isotropic displacement parameters (Å^2^)

**Table:**

	*x*	*y*	*z*	*U*_iso_*/*U*_eq_	
C1	0.4931 (2)	0.59395 (12)	0.15334 (9)	0.0254 (3)	
C2	0.5820 (2)	0.59443 (12)	0.22669 (9)	0.0245 (3)	
H2	0.5929	0.6582	0.2548	0.029*	
C3	0.6536 (2)	0.50227 (12)	0.25801 (9)	0.0246 (3)	
C4	0.6351 (2)	0.40751 (12)	0.21695 (9)	0.0277 (3)	
C5	0.5435 (2)	0.40639 (13)	0.14557 (9)	0.0302 (4)	
H5	0.5282	0.3421	0.1182	0.036*	
C6	0.4735 (2)	0.49990 (12)	0.11374 (9)	0.0283 (3)	
H6	0.4118	0.4992	0.0644	0.034*	
C7	0.7471 (2)	0.58042 (14)	0.37782 (10)	0.0363 (4)	
H7A	0.8078	0.6402	0.3568	0.054*	
H7B	0.8098	0.5613	0.4270	0.054*	
H7C	0.6236	0.6001	0.3839	0.054*	
C8	0.4221 (2)	0.69108 (13)	0.11667 (9)	0.0264 (3)	
H8	0.3633	0.6859	0.0669	0.032*	
C9	0.3561 (2)	0.86914 (13)	0.10164 (9)	0.0286 (3)	
H9A	0.2947	0.8417	0.0537	0.034*	
H9B	0.4520	0.9176	0.0894	0.034*	
C10	0.2231 (2)	0.92900 (14)	0.14407 (10)	0.0324 (4)	
H10A	0.1602	0.9819	0.1100	0.039*	
H10B	0.1329	0.8792	0.1598	0.039*	
C11	0.3670 (2)	0.93695 (13)	0.27651 (9)	0.0281 (3)	
H11	0.3487	0.8652	0.2889	0.034*	
C12	0.4516 (2)	1.10234 (13)	0.28397 (10)	0.0330 (4)	
H12	0.5030	1.1667	0.3031	0.040*	
C13	0.3652 (2)	1.08660 (13)	0.21491 (10)	0.0336 (4)	
H13	0.3445	1.1377	0.1760	0.040*	
C14	0.5274 (3)	0.98980 (16)	0.39957 (10)	0.0420 (4)	
H14A	0.4489	1.0207	0.4341	0.063*	
H14B	0.6457	1.0226	0.4081	0.063*	
H14C	0.5383	0.9138	0.4088	0.063*	
F1	−0.02389 (14)	0.28060 (9)	−0.01415 (7)	0.0477 (3)	
F2	0.17917 (17)	0.21418 (9)	−0.08507 (6)	0.0500 (3)	
F3	0.07180 (16)	0.11320 (8)	0.00376 (7)	0.0503 (3)	
F4	0.35699 (15)	0.17051 (10)	0.02179 (7)	0.0536 (3)	
F5	0.15258 (16)	0.23765 (10)	0.09197 (6)	0.0485 (3)	
F6	0.26139 (15)	0.33750 (8)	0.00272 (6)	0.0474 (3)	
N1	0.43423 (18)	0.78152 (10)	0.14719 (7)	0.0288 (3)	
N2	0.31202 (17)	0.98250 (10)	0.21108 (8)	0.0279 (3)	
N3	0.45170 (18)	1.00816 (11)	0.32170 (8)	0.0291 (3)	
O1	0.74720 (16)	0.49223 (9)	0.32760 (6)	0.0325 (3)	
O2	0.70614 (18)	0.31595 (9)	0.24688 (7)	0.0409 (3)	
H2A	0.7684	0.3288	0.2879	0.061*	
P1	0.16710 (6)	0.22501 (3)	0.00298 (2)	0.02858 (13)	

### Atomic displacement parameters (Å^2^)

**Table:**

	*U*^11^	*U*^22^	*U*^33^	*U*^12^	*U*^13^	*U*^23^
C1	0.0204 (7)	0.0258 (8)	0.0301 (8)	−0.0006 (6)	0.0031 (6)	0.0002 (6)
C2	0.0254 (7)	0.0209 (7)	0.0274 (8)	−0.0027 (6)	0.0036 (6)	−0.0027 (6)
C3	0.0240 (7)	0.0249 (8)	0.0249 (8)	−0.0043 (6)	0.0027 (6)	0.0006 (6)
C4	0.0300 (8)	0.0210 (7)	0.0316 (9)	−0.0009 (6)	0.0011 (6)	0.0015 (6)
C5	0.0332 (9)	0.0234 (8)	0.0334 (9)	−0.0014 (6)	0.0006 (7)	−0.0063 (7)
C6	0.0265 (8)	0.0299 (8)	0.0272 (8)	−0.0018 (6)	−0.0024 (6)	−0.0023 (7)
C7	0.0422 (10)	0.0370 (10)	0.0285 (9)	0.0013 (8)	−0.0018 (7)	−0.0083 (7)
C8	0.0234 (7)	0.0305 (8)	0.0248 (8)	0.0001 (6)	0.0005 (6)	0.0000 (6)
C9	0.0331 (8)	0.0259 (8)	0.0255 (8)	0.0028 (6)	−0.0024 (6)	0.0015 (6)
C10	0.0286 (8)	0.0317 (9)	0.0351 (9)	0.0054 (7)	−0.0052 (7)	−0.0021 (7)
C11	0.0296 (8)	0.0258 (8)	0.0291 (8)	−0.0015 (6)	0.0043 (6)	−0.0003 (7)
C12	0.0326 (9)	0.0247 (8)	0.0429 (10)	−0.0022 (7)	0.0101 (7)	−0.0038 (7)
C13	0.0357 (9)	0.0234 (8)	0.0427 (10)	0.0041 (7)	0.0087 (7)	0.0029 (7)
C14	0.0475 (11)	0.0507 (12)	0.0272 (9)	−0.0067 (9)	0.0018 (8)	−0.0051 (8)
F1	0.0338 (6)	0.0458 (7)	0.0599 (7)	0.0069 (5)	−0.0110 (5)	−0.0037 (5)
F2	0.0641 (8)	0.0555 (7)	0.0306 (6)	−0.0080 (6)	0.0059 (5)	−0.0050 (5)
F3	0.0612 (8)	0.0312 (6)	0.0591 (8)	−0.0099 (5)	0.0093 (6)	0.0020 (5)
F4	0.0404 (6)	0.0603 (8)	0.0592 (7)	0.0186 (6)	0.0011 (5)	0.0041 (6)
F5	0.0596 (7)	0.0563 (7)	0.0293 (6)	0.0083 (6)	0.0042 (5)	0.0001 (5)
F6	0.0505 (7)	0.0366 (6)	0.0513 (7)	−0.0149 (5)	−0.0111 (5)	0.0023 (5)
N1	0.0337 (7)	0.0258 (7)	0.0259 (7)	0.0046 (6)	−0.0006 (5)	0.0016 (6)
N2	0.0263 (7)	0.0256 (7)	0.0317 (7)	0.0044 (5)	0.0033 (5)	−0.0004 (6)
N3	0.0293 (7)	0.0290 (7)	0.0297 (7)	−0.0026 (6)	0.0064 (5)	−0.0031 (6)
O1	0.0436 (7)	0.0250 (6)	0.0267 (6)	0.0000 (5)	−0.0057 (5)	−0.0007 (5)
O2	0.0582 (9)	0.0214 (6)	0.0388 (7)	0.0025 (6)	−0.0149 (6)	−0.0013 (5)
P1	0.0299 (2)	0.0267 (2)	0.0283 (2)	0.00017 (17)	−0.00086 (16)	0.00051 (17)

### Geometric parameters (Å, °)

**Table:**

C1—C6	1.386 (2)	C10—N2	1.470 (2)
C1—C2	1.403 (2)	C10—H10A	0.9900
C1—C8	1.468 (2)	C10—H10B	0.9900
C2—C3	1.380 (2)	C11—N3	1.327 (2)
C2—H2	0.9500	C11—N2	1.327 (2)
C3—O1	1.3644 (18)	C11—H11	0.9500
C3—C4	1.406 (2)	C12—C13	1.343 (3)
C4—O2	1.3629 (19)	C12—N3	1.371 (2)
C4—C5	1.380 (2)	C12—H12	0.9500
C5—C6	1.393 (2)	C13—N2	1.379 (2)
C5—H5	0.9500	C13—H13	0.9500
C6—H6	0.9500	C14—N3	1.461 (2)
C7—O1	1.433 (2)	C14—H14A	0.9800
C7—H7A	0.9800	C14—H14B	0.9800
C7—H7B	0.9800	C14—H14C	0.9800
C7—H7C	0.9800	F1—P1	1.6008 (11)
C8—N1	1.269 (2)	F2—P1	1.5880 (11)
C8—H8	0.9500	F3—P1	1.5902 (11)
C9—N1	1.4618 (19)	F4—P1	1.5905 (11)
C9—C10	1.521 (2)	F5—P1	1.6101 (11)
C9—H9A	0.9900	F6—P1	1.5941 (11)
C9—H9B	0.9900	O2—H2A	0.8400
			
C6—C1—C2	119.47 (14)	N3—C11—N2	108.71 (15)
C6—C1—C8	118.84 (14)	N3—C11—H11	125.6
C2—C1—C8	121.68 (14)	N2—C11—H11	125.6
C3—C2—C1	119.96 (14)	C13—C12—N3	107.13 (15)
C3—C2—H2	120.0	C13—C12—H12	126.4
C1—C2—H2	120.0	N3—C12—H12	126.4
O1—C3—C2	125.97 (14)	C12—C13—N2	107.25 (15)
O1—C3—C4	113.93 (13)	C12—C13—H13	126.4
C2—C3—C4	120.09 (14)	N2—C13—H13	126.4
O2—C4—C5	119.19 (14)	N3—C14—H14A	109.5
O2—C4—C3	120.81 (14)	N3—C14—H14B	109.5
C5—C4—C3	120.00 (14)	H14A—C14—H14B	109.5
C4—C5—C6	119.71 (15)	N3—C14—H14C	109.5
C4—C5—H5	120.1	H14A—C14—H14C	109.5
C6—C5—H5	120.1	H14B—C14—H14C	109.5
C1—C6—C5	120.73 (14)	C8—N1—C9	116.33 (14)
C1—C6—H6	119.6	C11—N2—C13	108.20 (14)
C5—C6—H6	119.6	C11—N2—C10	125.74 (14)
O1—C7—H7A	109.5	C13—N2—C10	125.87 (14)
O1—C7—H7B	109.5	C11—N3—C12	108.71 (14)
H7A—C7—H7B	109.5	C11—N3—C14	125.50 (15)
O1—C7—H7C	109.5	C12—N3—C14	125.77 (14)
H7A—C7—H7C	109.5	C3—O1—C7	117.38 (12)
H7B—C7—H7C	109.5	C4—O2—H2A	109.5
N1—C8—C1	124.21 (14)	F2—P1—F3	90.34 (6)
N1—C8—H8	117.9	F2—P1—F4	91.38 (7)
C1—C8—H8	117.9	F3—P1—F4	90.37 (7)
N1—C9—C10	110.50 (14)	F2—P1—F6	90.07 (6)
N1—C9—H9A	109.6	F3—P1—F6	179.46 (7)
C10—C9—H9A	109.6	F4—P1—F6	89.96 (7)
N1—C9—H9B	109.6	F2—P1—F1	89.80 (7)
C10—C9—H9B	109.6	F3—P1—F1	89.88 (6)
H9A—C9—H9B	108.1	F4—P1—F1	178.80 (7)
N2—C10—C9	111.62 (13)	F6—P1—F1	89.78 (6)
N2—C10—H10A	109.3	F2—P1—F5	179.03 (7)
C9—C10—H10A	109.3	F3—P1—F5	90.06 (7)
N2—C10—H10B	109.3	F4—P1—F5	89.50 (6)
C9—C10—H10B	109.3	F6—P1—F5	89.52 (6)
H10A—C10—H10B	108.0	F1—P1—F5	89.32 (6)
			
C6—C1—C2—C3	1.8 (2)	N3—C12—C13—N2	0.31 (19)
C8—C1—C2—C3	−177.20 (14)	C1—C8—N1—C9	179.71 (14)
C1—C2—C3—O1	178.08 (14)	C10—C9—N1—C8	125.58 (16)
C1—C2—C3—C4	−1.0 (2)	N3—C11—N2—C13	0.40 (18)
O1—C3—C4—O2	0.3 (2)	N3—C11—N2—C10	175.73 (14)
C2—C3—C4—O2	179.53 (15)	C12—C13—N2—C11	−0.44 (19)
O1—C3—C4—C5	−179.91 (15)	C12—C13—N2—C10	−175.77 (15)
C2—C3—C4—C5	−0.7 (2)	C9—C10—N2—C11	−78.2 (2)
O2—C4—C5—C6	−178.65 (15)	C9—C10—N2—C13	96.36 (19)
C3—C4—C5—C6	1.6 (3)	N2—C11—N3—C12	−0.20 (19)
C2—C1—C6—C5	−1.0 (2)	N2—C11—N3—C14	178.01 (15)
C8—C1—C6—C5	178.10 (15)	C13—C12—N3—C11	−0.08 (19)
C4—C5—C6—C1	−0.8 (3)	C13—C12—N3—C14	−178.29 (16)
C6—C1—C8—N1	−179.14 (16)	C2—C3—O1—C7	10.9 (2)
C2—C1—C8—N1	−0.1 (2)	C4—C3—O1—C7	−169.95 (15)
N1—C9—C10—N2	66.34 (18)		

### Hydrogen-bond geometry (Å, °)

**Table:**

*D*—H···*A*	*D*—H	H···*A*	*D*···*A*	*D*—H···*A*
O2—H2A···O1	0.84	2.20	2.6589 (16)	114
O2—H2A···N1^i^	0.84	2.48	3.1584 (18)	139
O2—H2A···F2^ii^	0.84	2.49	3.0487 (18)	125

Symmetry codes: (i) −*x*+3/2, *y*−1/2, −*z*+1/2; (ii) *x*+1/2, −*y*+1/2, *z*+1/2.
